# Human exposure to pollutant emissions from brick kilns and their association with ocular health and neurotoxic symptoms: a pilot study in the Refugio Brickmaking Area, León, Mexico

**DOI:** 10.3389/fpsyt.2025.1554101

**Published:** 2025-06-13

**Authors:** Paula Karolina Pedroza Carrillo, Anuar Salazar-Gómez, Ana Mariela Jimenez Alcala, Daniela del Rocío Nilo Olmos, Blanca Perez Perez, Valeria Guadalupe Rocha Villa, Ana Laura Martínez-Rodríguez, Stéphanie C. Thébault, Luis Fernando Hernández-Zimbrón

**Affiliations:** ^1^ Laboratorio de Investigación interdisciplinaria, Área de Optometría, Escuela Nacional de Estudios Superiores, Unidad León, Universidad Nacional Autónoma de México (UNAM), Guanajuato, Mexico; ^2^ Clínica de Optometría, Escuela Nacional de Estudios Superiores, Unidad León, Universidad Nacional Autónoma de México (UNAM), Guanajuato, Mexico; ^3^ Laboratorio de Investigación Traslacional en Salud Visual D-13, Instituto de Neurobiología, Universidad Nacional Autónoma de México (UNAM), Queretaro, Mexico

**Keywords:** brick kilns, air pollution, neurotoxicity symptoms, ocular health, questionnaire, community study

## Abstract

León, Guanajuato, Mexico, like many other places in the world, faces significant air pollution due to emissions from artisanal brick kilns. If prolonged exposure to these pollutants has been associated with neurotoxicity symptoms and potential risks of neurodegenerative diseases, their effects on ocular health remain poorly understood. Therefore, this study evaluated the visual health, prevalence of neurotoxicity symptoms and mental health conditions, as well as potential associations in residents near the Refugio Brickmaking Region, in León, Guanajuato, Mexico. A cross-sectional study was conducted on thirteen participants working or living in brick kilns and thirteen control participants. Clinical evaluations included vital signs, uncorrected distance visual acuity (UDVA), and refraction test. A Likert scale modified version of the Q16 neurotoxicity questionnaire was used to evaluate neurotoxic symptoms and mental health conditions were identified through the clinical history. Neurotoxicity scores were compared between groups using the Mann Whitney U test, while the Chi-square test was used to assess the association between working or living in brick kilns and the prevalence of ocular and neurotoxic symptoms. The groups were paired in age (44.64 ± 16.4 vs. 52.38 ± 18.4 years-old for exposed and control groups, respectively), The alleged exposed group had an average age of 44.64 years, compared to 52.38 years in the controls). Six ocular symptoms were prevalent among participants working or living in brick kilns: foreign body sensation, blurred vision, itchy eyes, watery eyes, photophobia, and decreased visual acuity. These participants also had diminished binocular uncorrected distance visual acuity (UDVA ≥ 0.3 logMAR), and increased prevalence of myopia. Also, a positive association was found between working or living in brick kilns and reported anxiety/depression, irritability, and insomnia. These data clearly demonstrated a deterioration in eye health, in addition to an increased prevalence of neurotoxicity symptoms, in relation to working or living in brick kilns, which highlights the need for stricter regulations to safeguard workers and residents in high-risk areas.

## 1 Introduction

The brick kiln industry is an important economic sector in many countries with over 1000 billion bricks produced annually worldwide, making Asia the world’s largest brick producer (1). In Latin America, there are approximately 45,000 brick producers, with Brazil and Mexico being the largest producers in the region (2). It is estimated that 30-50% of bricks produced in Latin America come from artisanal procedures in establishments that operate under unregulated conditions (3). Mexico has approximately 17,000 brick kilns operating with artisanal methods, and Guanajuato state is home to a large part of this sector (4).

The brick-making process relies on traditional technologies with adverse environmental and social impacts, especially high pollutant emissions (5). Exposure to air pollutants from brick kilns causes acute and chronic effects on human health, notably affecting the pulmonary system (6, 7), particularly in workers and adolescents constantly exposed to brick-kiln emissions in Mexico (8–10). The main environmental pollutants from brick kilns include particulate matter with aerodynamic diameter ≤2.5 μm (PM_2.5_), polycyclic aromatic hydrocarbons (PAHs), nitrogen dioxide (NO_2_), sulfur dioxide (SO_2_), and carbon monoxide (CO), among others (11). Studies in Guanajuato state have shown elevated levels of PM_2.5_ in the air (11), and women residing in brick-manufacturing areas have been found to be exposed to PAHs (11, 12).

In addition to affecting the pulmonary system, air pollution can also impact other critical organs and systems in the human body (12). Exposure to PM_2.5_ has been linked to an increased risk of neurotoxic manifestations and ocular problems (13–15). These fine particulate matter pollutants can reach the brain directly through the nasal mucosa-olfactory nerve pathway, traveling to the olfactory bulb. Additionally, PM_s_ can enter the central nervous system through the eye-nose-brain route via the nasolacrimal ducts (15–17).

Despite the documented neurotoxic and ocular effects of exposure to environmental pollutants, research on eye diseases and neurotoxic manifestations in populations living near brick manufacturing sites remains limited, especially in Mexico, where only two studies have been published (7, 18).

**Figure 1:**
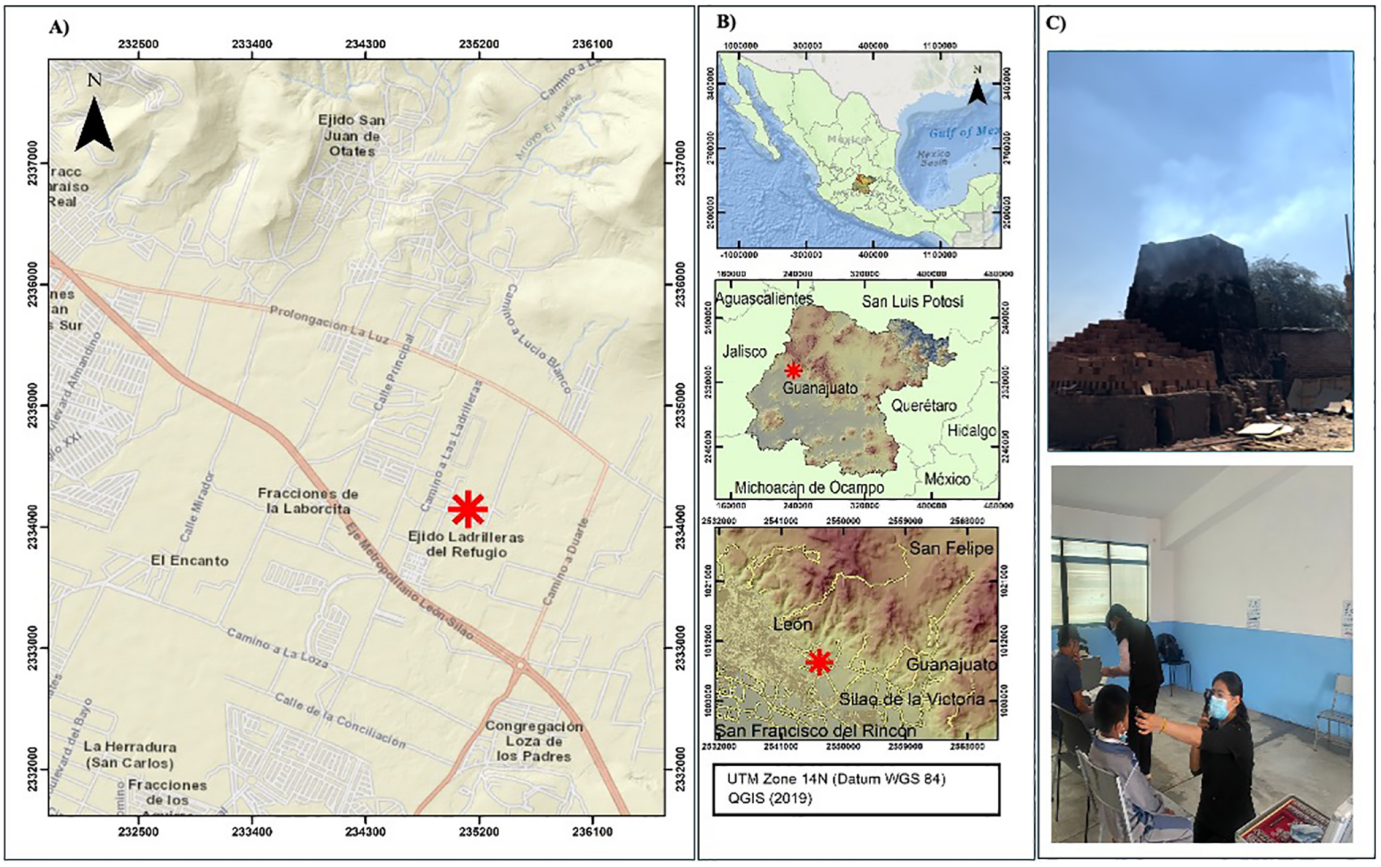
**(A)** shows the Ladrilleras del Refugio area, with a red star marking the brick kilns located at the specified coordinates (21.089248, -101.549602). **(B)** is an inset series of the central region of Mexico, highlighting the location of León, Guanajuato, where the study took place. The study area is located in Universal Transverse Mercator (UTM) Zone 14N, at coordinates 235115.46 m E, 2334146.69 m N (Datum WGS 84), which correspond to 21.0892° N, 101.5496° W in geographic coordinates (QGIS Development Team (2019) QGIS Geographic Information System. Open Source Geospatial Foundation Project). **(C)** displays images of the brick kilns where the “firing” process of the bricks takes place, with visible smoke curtains produced regularly (top). illustrates how the ocular health evaluations were conducted by optometrists (bottom).

Ladrilleras del Refugio is a rural community located near León, one of the major cities in the state of Guanajuato, Mexico (**Figure 1**). According to data from the National Institute of Statistics and Geography (INEGI), of Mexico, the community had a population of approximately 1,815 residents as of 2019. Situated at an altitude of 1,862 meters, the area is primarily known for its artisanal brick production, which plays a significant role in the local economy (19). Given these conditions, we designed a pilot study to evaluate visual health, the prevalence of self-reported symptoms of neurotoxicity and mental health conditions among residents living near the Refugio Brickmaking Region, León, Guanajuato. Additionally, we investigated whether an association exists between exposure (living near the brick kilns) and the presence of ocular symptoms, neurotoxicity, or neurological symptoms.

## 2 Methods

### 2.1 Study design

A cross-sectional pilot case-control study was conducted in the Refugio Brickmaking Region, León, Guanajuato, Mexico, from November 15, 2022, until April 30, 2023. The study protocol was approved by Research Ethics Committee of the Escuela Nacional de Estudios Superiores, Unidad León, UNAM (CEI_22_06_S21) and conducted in accordance with Good Clinical Practice and performed according to the Declaration of Helsinki. Written informed consent was obtained from all participants.

### 2.2 Participants

A purposively selected sample of 26 participants (aged ≥ 18 years) was recruited for this protocol. Thirteen participants who work or live near the brick kiln areas (exposed group) were recruited from the visual health brigade in Camino a Ladrilleras, Carretera León-Duarte, León, México. A control group of thirteen participants was selected from those not exposed to the brick kiln area. All participants provided a detailed medical history, including demographic data, lifestyle factors (such as smoking and alcohol consumption), and ocular health history, as well as self-reported anxiety or depression. Height, body weight, and blood pressure were measured, and body mass index (BMI) was calculated as weight in kilograms divided by height in meters squared (kg/m²).

Participants underwent a comprehensive clinical eye examination including uncorrected distance visual acuity (UDVA) and refraction test, following using standardized protocols (20). Visual acuity records ranged from 0 logMAR units (Snellen equivalent 20/20) to 1.3 logMAR units (Snellen equivalent 20/400), with lower values indicating better visual acuity (21). Refractive errors were classified as follows: myopia was defined as ≤-0.25 D, and hyperopia as ≥+0.25 D, and astigmatism as a cylinder of ≤-0.25 D (22).

### 2.3 The neurotoxicity symptoms questionnaire (Q16)

A self-administered modified Q16 questionnaire was used to evaluate neurotoxic symptoms (23). The questionnaire was modified by including sixteen questions that employed a 5-point Likert scale, categorizing responses into ranges: 6 to 32 for low, 33 to 48 for medium-low, 49 to 64 for medium, and 65 to 80 for high, to provide more information about neurotoxicity symptoms according to Jiménez- Barbosa et al. (24).

### 2.4 Data and statistical analysis

Descriptive statistics were presented as means with standard deviations (SD) for continuous variables and as percentages for categorical variables. UDVA data were converted to the log of the minimum angle of resolution (logMAR) for statistical analyses. Non-parametric comparisons between groups were conducted using the Mann-Whitney U test, with statistical significance set at *p* < 0.05.

The Chi-square test was used to evaluate the association between pollutant exposure and the presence of ocular and neurotoxic symptoms. A contingency table was constructed to determine whether the distribution of symptoms significantly differed between exposed and non-exposed participants. This test assessed whether pollutant exposure was associated with a higher prevalence of self-reported symptoms compared to what would be expected by chance. A *p*-value of <0.05 was considered indicative of a statistically significant association. All statistical analyses were conducted using GraphPad Prism^®^ version 9.0.1 for Windows (California, USA).

## 3 Results

### 3.1 Demographic information and lifestyle factors

The demographic characteristics of participants are presented in **Table 1**. The mean age of participants was 48.96 ± 16.40 years (range: 21–76 years, women:men 1:1. More than half (84.6%) of participants exposed to the brick manufacturing site in Ladrilleras del Refugio have worked for between 6 and 60 years in brick kilns, for at least six hours a day, every day (5–7 days).

**Table 1:** Demographic characteristics and lifestyle factors of the participants included in the study.

Characteristics	Total (n=26)	Non-exposed (n=13)	Exposed (n=13)
Mean age in years (± SD)	48.96 ± 16.40	52.23 ± 15.25	46.85 ± 13.75
Sex Female Male	1313	58	85
				Body mass index (kg/m^2^) (± SD)	25.44 ± 4.76	28.1 ± 7.2	28.73 ± 6.94
Systolic blood pressure (mm/Hg) (± SD)	123.6 ± 22.7	123.31 ± 14.43	125.14 ± 22.99
Cigarette smoking (%)	11.5	15.4	7.7
Alcohol consumption (%)	26.9	46.2	7.7
Diabetes	9	6	3
Years of exposure; median (± SD)	–	–	29.42 ± 18.02; 30

SD= standard deviation. Data are presented as mean ± SD or percentages where applicable.

**Table 1** Demographic information and lifestyle factors of the study participants, including both non-exposed and exposed groups.

If seven participants reported general brick production activities, three were involved in brickmaking, one worked directly in the kiln, and two were not working directly in brick production, all were living within the facilities of the brickmaking complex and were therefore considered exposed. 81.82% of these brick kiln workers did not have health care services. Alcohol consumption (46.2%) and smoking (15.4%) were more prevalent in the control group than in the alleged exposed group (26.9% and 11.5% for alcohol consumption and smoking, respectively).

The control group consisted of individuals working in professions such as teachers, homemakers, and skilled trades, including gardening, carpentry, and baking. These occupations typically do not involve significant exposure to air contaminants. While differences between groups may be influenced by factors beyond pollutant exposure, the nature of the occupations in the control group reduces the likelihood of occupational exposure to air contaminants.

### 3.2 Visual acuity, refractive status, and other ocular findings

Six prevalent symptoms related to eye problems were identified in the alleged exposed group, including foreign body sensation in twelve participants (92.3%), blurred vision and itchy eyes in ten participants (76.92%), watery eyes and eye discharge in nine of them (69.23%), photophobia and eye pain, and headache in eight of them (61.53%), and decreased visual acuity and nyctalopia in seven of them (53.84%). We found that decreased visual acuity and blurred vision and itchy eyes, were not more prevalent in the population supposedly exposed to pollutants from brick kilns (**Table 2**). Also, the association of exposure to environmental pollutants and more prevalent ocular symptomatology was evaluated, finding a positive association assessed using Chi-square (χ²) for the following symptoms: Foreign body sensation (18.62, p=0.0001), Watery eyes and eye discharge (7.72, p=0.005), Photophobia (3.93, p=0.047), Eye pain (8.32, p=0.003), Headache (8.32, p=0.003), and Nyctalopia (9.57, p=0.002). In contrast, no positive association was found for Decreased visual acuity (2.60, p=1.612) and Blurred vision and itchy eyes (2.60, p=1.612) (**Table 2** **).**

**Table 2:** Association of exposure to environmental pollutants and more prevalent ocular symptomatology.

Symptom/Condition	Nonexposed (n=13)	Exposed (n=13)	Chi-square value	p-value
Foreign body sensation	1 (07.69%)	12 (92.30%)	18.62	<0.0001
Blurred vision and itchy eyes	6 (46.15%)	10 (76.92%)	2.60	1.612
Watery eyes and eye discharge	2 (15.38%)	9 (69.23%)	7.72	0.005
Photofobia	3 (23.07%)	8 (61.53%)	3.93	0.047
Eye pain	1 (07.69%)	8 (61.53%)	8.32	0.003
Headache	1 (07.69%)	8 (61.53%)	8.32	0.003
Decreased visual acuity	3 (23.07%)	7 (53.84%)	2.60	1.612
Nyctalopia	0 (0.00%)	7 (53.84%)	9.57	0.002

**Table 2** summarizes the association between exposure to environmental pollutants and ocular symptomatology. Chi-square values greater than 3.84 and p-values less than 0.05 indicate statistically significant associations. The results for ‘Blurred vision and itchy eyes’ and ‘Decreased visual acuity’ were not statistically significant (p ≥ 0.05).

In another way, while 91.6% of controls exhibited a binocular uncorrected distance visual acuity (UDVA) of 20/25 Snellen (0.1 logMAR) or better, 50% of the participants allegedly exposed exhibited a binocular uncorrected distance visual acuity (UDVA) of 20/40 Snellen (0.3 logMAR) or worse. In both eyes, 50% of the exposed patients showed an UDVA of 20/30 Snellen (0.18 logMAR) (**Table 3**). Of the twelve participants exposed, myopia occurred in nine (69.23%), hyperopia in two (15.38%), and astigmatism in eleven (84.61%). In contrast, myopia occurred in four participants (33.33%), hyperopia in six of them (46.5%), and astigmatism in eight (61.53%) of the control participants.

**Table 3:** Comparison of visual acuity between non-exposed and exposed participants.

Parameters	Non-exposed (n=13)	Exposed (n=13)	*p*-value
Left eye visual acuity [logMAR mean (± SD)] (range)	0.09 ± 0.10 (0-0.3)	0.47 ± 0.42 (0.18-1.3)	p<0.05
Right eye visual acuity [logMAR mean (± SD)] (range)	0.14 ± 0.11 (0-0.3)	0.47 ± 0.39 (0.18-1.3)	p<0.05
Binocular uncorrected distance visual acuity [logMAR mean (± SD)] (range)	0.03 ± 0.06 (0-0.18)	0.46 ± 0.42 (0.18-1.3)	p<0.01

logMAR= logarithm of the minimum angle of resolution. SD= standard deviation. Data are presented as mean ± SD.

**Table 3** describes the comparison of visual acuity of both groups. Exposed participants shown more refractive errors in both eyes compared to non-exposed group. There were statistically significant differences across both groups.

### 3.3 Neurotoxicity symptoms

The neurotoxicity levels were medium-low (36.08 ± 14.9) for the participants working or living in brick kilns, while the control group had a low neurotoxicity level (28.62 ± 7.78) (**Table 4**). Two of the most common responses by the alleged exposed group were: “*I feel abnormally tired”* and *“I often wake up and then have problems getting back to sleep again.”*. The calculated U value was -32, while the critical value of U from the table at a significance level of α = 0.01 is 39. Since the calculated U value of -32 is less than the critical value of 39, the null hypothesis was rejected, indicating that there was a statistically significant difference in neurotoxicity scores between the alleged exposed and control groups.

**Table 4:** Comparison of neurotoxicity scores between exposed and non-exposed participants.

Symptom	Nonexposed (n=13)	Exposed (n=13)	U-value	*p*-value
Neurotoxicity score (± SD)*	Low (28.62 ± 7.78)	Medium-Low (36.08 ± 14.9)	32	0.2924

SD= standard deviation. Data are presented as mean ± SD.

*p*-value obtained from the Mann-Whitney U test comparing the non-exposed group and exposed groups.

**Table 4** presents the neurotoxicity scores of participants in the non-exposed and exposed groups, categorized as Low (28.62 ± 7.78) and Medium-Low (36.08 ± 14.9), respectively. A Mann-Whitney U test was performed, yielding a U-value of 32 and a p-value of 0.2924. Data are presented as mean ± standard deviation (SD).

Additionally, 61.5% of the participants working or living in brick kilns reported anxiety or depression versus 15.3% in the control group. The Chi-square test yielded a value of 4.06 (p = 0.044), indicating that there is a significant association between working or living in brick kilns and anxiety and depression (**Table 5**). Similarly, irritability, reduced sensation in arms and legs, and insomnia were found to associate with working or living in brick kilns (**Table 5**). Our analysis also showed a lack of association between the existence of neurological history and working or living in artisanal brick factories (**Table 5**). These data do not indicate an association between occupational exposure to brick kiln pollutants and mental health outcomes.

**Table 5:** Association of exposure to environmental pollutants and individual neurotoxically symptoms (Q16) or self-reported neurological disease.

Symptom/Condition	Nonexposed (n=13)	Exposed (n=13)	Chi-square value	p-value
Reported anxiety/depression	2 (15.3%)	8 (61.5%)	5.850	0.015
Irritation	1 (7.69%)	8 (61.5%)	8.32	0.039
Reduced sensation in arms and legs	1 (7.69%)	5 (38.4%)	3.46	0.062
Insomnia	3 (23.07%)	8 (61.5%)	3.93	0.047
Neurotoxicity (Q16+)	4 (30.76%)	7 (53.84%)	1.418	0.233
Suffer from any neurological disease	2 (15.38%)	0 (0.0%)	2.167	0.14

**Table 5** summarizes the association between exposure to environmental pollutants and neurotoxic symptoms or neurological disease, comparing the exposed and non-exposed groups. Chi-square values and p-values indicate statistically significant associations for *reported anxiety/depression*, *irritability*, and *insomnia*, all of which were more prevalent in the exposed group. However, results for positive neurotoxicity obtained from the Q16 questionnaire and self-reported cases of “suffering from any neurological disease” were not statistically significant.

## 4 Discussion

The impact of exposure to air pollutants produced by brick-making on ocular and mental health has been poorly documented. Our data showed a higher prevalence of ocular symptoms including foreign body sensation, blurred vision and itchy eyes, watery eyes and eye discharge, photophobia, eye pain, headache, decreased visual acuity and nyctalopia. Additionally, myopia was more common among individuals working or living near brick kilns. Furthermore, neurotoxicity scores were higher in the alleged exposed group compared to the control group. Living or working in brick kilns was also associated with a higher prevalence of self-reported symptoms, including irritability, anxiety, depression, and insomnia.

Mexico is one of the biggest brick producers in Latin America, and many brick kilns operate using artisanal methods. Our data add to previous reports showing that brick kiln workers are exposed not only to physical risk factors, but also to increased risk of health problems, including respiratory, musculoskeletal, gastrointestinal, reproductive, dermic, psychosocial disorders or complaints (6, 12, 25), as well as eye irritation (26). Indeed, people exposed to the brick manufacturing site in Ladrilleras del Refugio presented ocular symptomatologies, as our results show. The significant associations found between exposure to environmental pollutants and symptoms such as foreign body sensation, watery eyes and eye discharge, photophobia, eye pain, headache, and nyctalopia suggest that pollutants released in these environments have a direct impact on ocular health. These findings align with previous studies that report increased ocular irritation and discomfort in populations exposed to airborne contaminants, particularly in industrial and highly polluted areas (26).

The strong association observed for foreign body sensation, and watery eyes with eye discharge may indicate that particulate matter and chemical irritants present in the brick kiln environment induce chronic irritation and excessive tear production as a protective mechanism. Additionally, the association with photophobia and eye pain suggests that these pollutants may cause inflammatory responses or affect the ocular surface, leading to increased sensitivity to light and discomfort.

Furthermore, the significant relationship between exposure and headache and nyctalopia raises concerns about the broader neurological and visual implications of prolonged exposure to these environmental hazards. These symptoms could be indicative of systemic effects beyond ocular irritation, potentially involving the central nervous system or retinal function.

In contrast, the lack of a significant association for decreased visual acuity, and blurred vision with itchy eyes suggests that, while pollutants cause acute ocular discomfort, they may not be directly linked to long-term visual impairment within the studied population. However, further longitudinal studies are needed to determine whether continuous exposure could lead to progressive visual decline over time.

Foreign body sensation is the primary symptom associated with exposure to environmental pollutants such as PM, PAHs, NO_2_, and SO_2_ (6, 12). PM_2.5_, PAHs, NO_2,_ and CO are the primary environmental pollutants emitted from brick kilns (6, 12). The concentration of PM_2.5_ registered in the brickyard of the community of Yerbabuena, Guanajuato, exceeds the maximum permissible limits established in Mexican environmental and regulations (27). In addition to eye irritation, we observed reduced visual acuity and increased incidence of myopia among individuals working or living in brick kilns. This finding aligns with prior studies demonstrating associations between pollutant exposure and refractive errors (28, 29). Specifically, exposure to PM_2.5_ and NO_2_ has been linked to an increased risk of myopia (29).

Moreover, exposure to environmental PM_2.5_ has been increasingly linked to a higher risk of neurotoxic manifestations (6). In this line, we showed that the group working or living in brick kilns showed higher neurotoxicity scores compared to the control group. Specifically, the medium-low neurotoxicity scores in the alleged exposed group to brick kiln pollutants was significantly different from the low neurotoxicity scores in the control group. Taken together with the positive association detected between working in brick kilns and reduced limb sensitivity, these findings strongly suggest a neurotoxic impact of brick kiln pollutants. To our knowledge, this is the first study to report self-reported neurotoxic symptoms in individuals working or living in brick kilns using a modified Q16 questionnaire, originally designed as a screening tool for neurotoxic symptomatology in individuals exposed to solvents. Generally, brick making does not require the use of solvents, but this activity emits both gaseous and particulate pollutants (6), reason for which we used it to assess the relationship between work or live near the brick kiln areas (called exposure) and neurotoxic symptoms and exposure and ocular symptomatology.

Previous research has shown that occupational exposure to agrochemicals in workers with neurotoxic signs is associated with deficits in both low and high visual perception (30). Similarly, abnormal pupil size in agricultural workers exposed to pesticides, has been linked to neurotoxicity, as evidenced by associated symptomatology and altered cholinesterase levels (31–33). These findings highlight the nexus between environmental toxins and neurotoxicity. While our study explored this relationship, we did not find a statistically significant association between exposure and neurotoxicity, highlighting the need for further research to clarify this potential link.

Additionally, we found important associations between exposure to brick kiln pollutants and mental health conditions that included anxiety, depression, irritation, and insomnia. Before commenting that these four mental health conditions are closely linked, it is important to note that our control group is not exempt from some of these conditions. Two control participants reported anxiety or depression. However, in theory, this potential bias should hinder the achievement of a significant difference. As this has been obtained, we can conclude that the association between working or living in a brick kiln and depression and anxiety is all the stronger. In contrast, the lack of association between working or living in brick kilns and neurological diseases is very likely due to the existence of a neurological history in two of the control cases. We recognize this limitation not in the design of our pilot study, but in the size of our sample. Indeed, mental health disorders and neurological conditions can result(arise) from a variety of factors beyond exposure to environmental pollutants, such as socio-economic condition, stress, or others.

Our observations showing an association between anxiety, depression, irritability, insomnia, and working or living in brick kilns are consistent with well-established relationships between these mental health conditions. Anxiety and depression are among the chief causes of insomnia, and inversely, sleep disturbance increases the risk of depression and/or anxiety (34). Irritation caused by exposure to particulate matter and toxic gases has been related to chronic stress and diminished quality of life (33). Furthermore, long-term physical discomfort from irritation can trigger stress responses, increasing the risk of psychiatric symptoms such as irritability, anxiety, and depressive states, especially in underserved communities already struggling with socio-economic challenges (35). In this sense, brick kiln workers often engage in arduous activities, which contributes to dangerous working and living conditions that demand not only physically but also emotionally.

These positive associations observed in the alleged exposed group emphasize the impact of air pollutants on mental health and well-being. Air pollution has been shown to disrupt the central nervous system, contributing to systemic inflammation and oxidative stress that impair sleep quality (36). Insomnia, in turn, is strongly associated with the onset and worsening of depression and anxiety, as well as impairments in cognitive function and emotional regulation (37). In fact, exposure to petroleum-derived hydrocarbons is associated with self-reported sleep disturbances and neurotoxicity symptoms (38–40). It is important to note that sleep disorders of brick kiln workers can also be related to other factors, such as the noisy environment in brick kiln areas (41, 42).

As for the potential mechanisms behind mental health conditions and brick kiln pollutants, prior research show that prolonged exposure to pollutants like PM_2.5_, NO_2_, and combustion by products associates with neuroinflammatory processes and oxidative stress, leading to disruptions in neurotransmitter systems, particularly serotonin and dopamine, which are critical for mood regulation (31, 36). These mechanisms have been proposed to contribute to depressive symptoms in populations exposed to environmental pollution in both urban and occupational settings (38). Despite the existence of Mexican laws to protect the environment against air pollutants, the observations of our study and others show the disease conditions that brick kiln workers and people who live in these facilities face, probably due to continuous exposure to air pollutants. We call for the protection measures set out in the law to be applied.

The primary limitation of this study is its small sample size, typical of pilot studies, which restricts the generalizability of the findings, but also make some of our observations uncertain, particularly the lack of association between working and living in brick kilns and neurotoxicity or neurological diseases. More details about the demographic and occupational characteristics of the alleged exposed and control groups are required to refine our risk factor analysis. Most importantly, the lack of direct pollutant concentration measurements limited our ability to input neurotoxic and ocular symptoms to brick kiln pollutants. Long-term implications of pollutant exposure should also be studied, particularly on the potential progression to neurodegenerative diseases. The type of eye problems to look for could also be expanded in future studies, as previous research on children living near brick kilns reported scleral melanosis and conjunctival changes (43).

## 5 Conclusions

Our results suggest that working or living in brick kilns negatively impacts ocular health and may be associated with neurotoxic effects. Additionally, we found an increased prevalence of psychiatric symptoms, highlighting the need for further mechanistic studies and long-term research on the ocular and neurological effects of brick kiln exposure. These findings also underscore the urgent need for judicial authorities to enforce existing environmental regulations aimed at protecting vulnerable communities living and working near brick kilns. Moreover, ensuring these populations have access to adequate healthcare is essential to mitigate potential health risks.

## Data Availability

The raw data supporting the conclusions of this article will be made available by the authors, without undue reservation.
